# The Adenosine Deaminase Gene Polymorphism Is Associated with Chronic Heart Failure Risk in Chinese

**DOI:** 10.3390/ijms150915259

**Published:** 2014-08-28

**Authors:** Hai-Rong He, Yuan-Jie Li, Gong-Hao He, Ya-Jun Wang, Ya-Jing Zhai, Jiao Xie, Wei-Peng Zhang, Ya-Lin Dong, Jun Lu

**Affiliations:** 1 Department of Pharmacy, the First Affiliated Hospital of Medical College, Xi’an Jiaotong University, Xi’an 710061, China; E-Mails: hehairong19891989@126.com (H.-R.H.); a3093896@163.com (Y.-J.Z.); xiejiao159753@163.com (J.X.); zhangwp0126@126.com (W.-P.Z.); 2 Department of Human Anatomy, Histology and Embryology, Medical College, Xi’an Jiaotong University, Xi’an 710061, China; E-Mail: liyj2010@mail.xjtu.edu.cn; 3 Department of Pharmacy, Kunming General Hospital of Chengdu Military Region, Kunming 650032, China; E-Mail: gonghow@hotmail.com; 4 Physical Examination Department, the First Affiliated Hospital of Medical College, Xi’an Jiaotong University, Xi’an 710061, China; E-Mail: 13709199383@163.com

**Keywords:** ADA, adenosine, chronic heart failure, SNP, rs452159

## Abstract

Adenosine (Ado) is an important cardioprotective agent. Since endogenous Ado levels are affected by the enzyme Ado deaminase (ADA), polymorphisms within the *ADA* gene may exert some effect on chronic heart failure (CHF). This study applied a case-control investigation to 300 northern Chinese Han CHF patients and 400 ethnicity-matched healthy controls in which nine single-nucleotide polymorphisms (SNPs) of ADA were genotyped and association analyses were performed. Odds ratios (ORs) with 95% confidence intervals (CI) were used to assess the association. Overall, rs452159 polymorphism in *ADA* gene was significantly associated with susceptibility to CHF under the dominant model (*p* = 0.013, OR = 1.537, 95% CI = 1.10–2.16), after adjustment for age, sex, and traditional cardiovascular risk factors. No difference in genotype distribution and allele frequency for the rs452159 according to the functional New York Heart Association class was found. Furthermore, the values of left ventricular ejection fraction, left-ventricle end-diastolic diameter or left-ventricle end-systolic diameter did not differ significantly among the different rs452159 genotype CHF patients. Although further studies with larger cohorts and other ethnicities are required to validate the conclusions, the findings of this study potentially provide novel insight into the pathogenesis of CHF.

## 1. Introduction

Chronic heart failure (CHF) is a common and complex syndrome that affects patients worldwide. The associated mortality rate remains high, although the prognosis and quality of life of CHF patients have greatly improved over the last decade. It has been reported that 40% of patients with CHF die after hospital admission or within 1 year after they are admitted [1,2].

Adenosine (Ado) is an endogenous nucleoside that has cardioprotective properties, modulating numerous physiological processes including coronary blood flow [3,4]. There is evidence that the adenosinergic system is essential to the mediation of intrinsic cardioprotection and for determining myocardial resistance to insult [5]. There is also experimental evidence that endogenous Ado can optimize the relationship between myocardial energy production and consumption, thus improving the body’s utilization rate of oxygen [6,7]. Recent studies have pointed out that Ado can reduce the end-diastolic pressure and increase the descending rate of left-ventricle pressure to improve cardiac systolic and diastolic function [8]. In addition, this nucleoside exerts electrophysiological effects on supraventricular tissue that lead to a reduced heart rate [9]. The protective effect of Ado in failing hearts is thought to be attributable to its anti-inflammatory, antiapoptotic, antiadrenergic, and antioxidant properties [10]. Furthermore, Ado can protect the heart from ischemia-reperfusion injury and cardiac remodeling, thus preventing the progress of heart failure [11].

Endogenous Ado levels can be affected by Ado deaminase (ADA), which catalyzes the formation of inosine from Ado. A recent analysis of gene-modified mice has confirmed these findings with the pharmacological inhibition of Ado metabolism, showing that inhibition of ADA increases Ado levels, thereby affecting its cardioprotective role [8,11,12,13,14]. ADA activity could be affected by gene polymorphisms *in vivo*. It is therefore rational to hypothesize that single-nucleotide polymorphisms (SNPs) in the ADA gene (*ADA*) could influence the activity of the protein, and further affect the susceptibility to and progress of CHF. To the best of our knowledge, this hypothesis has not been investigated previously, and hence the present study tested it by evaluating the relationship between certain tag SNPs of *ADA* and susceptibility to CHF among a northern Chinese Han population, with a view to providing some insight into the prevention and individualized treatment of CHF.

## 2. Results

### 2.1. Population Characteristics

The demographic and clinical characteristics of the study populations are reported in Table 1. The prevalence of traditional cardiovascular risk factors was significantly higher in CHF patients than in the control group (*p* < 0.05).

**Table 1:** Demographic and clinical characteristics of study populations.

Factors		Chronic Heart Failure (CHF)	Controls	*p* Value
Age (years)		61.41 ± 12.51	60.33 ± 8.56	0.2 ^a^
Males/females		175/125	211/189	0.14 ^b^
Hypertension		172 (57.33%)	122 (30.5%)	<0.001 ^b^
Dyslipidemia		84 (28%)	81 (20.25%)	0.0168 ^b^
Diabetes		104 (34.67%)	67 (16.75%)	<0.001 ^b^
Smoking habit		91 (30.33%)	62 (15.5%)	<0.001 ^b^
CAD		180 (60%)	–	
ICDM		76 (25.33%)	–	
HC		9 (3%)	–	
Other diagnose		35 (11.67%)	–	
LVEF ≤ 40%		207 (69%)	–	
NYHA class	II	94 (31.33%)	–	
	III	117 (39%)	–	
	IV	89 (29.67%)	–	

Abbreviations: CAD, coronary artery disease; ICDM, idiopathic dilated cardiomyopathy; HC, hypertensive cardiomyopathy; LVEF, left ventricular ejection fraction; NYHA, New York Heart Association class; ^a^ *p* values were calculated by separate variance estimation *t*-test as variance between the two group is not neat; ^b^ *p* values was calculated from two-sided χ^2^-test.

### 2.2. Association between Tag Single-Nucleotide Polymorphisms (SNPs) and Susceptibility to Chronic Heart Failure (CHF)

The distributions of the genotype and allele frequencies of the nine SNPs of the ADA gene in CHF patients and healthy controls are listed in Table 2. The distributions of all of the genotypes except rs73598374 for the healthy controls were in accordance with HWE. The rs452159 variant was associated with a significant predisposition to CHF under the dominant and additive models of inheritance. However, the rs8119756 variant was associated with a significant predisposition to CHF only under the dominant model. In addition, the A allele of rs452159 was found to be a risk factor for CHF. None of the other tag SNPs was associated with the susceptibility to CHF (Table 2). After adjustment for age, sex, and traditional cardiovascular risk factors, only the rs452159 SNP significantly and independently influenced the susceptibility to CHF under the dominant model (Table 3).

**Table 2:** Genotype distribution of the eight tag single-nucleotide polymorphisms (SNPs) and their associations with the risk of chronic heart failure under different contrast models.

Genotype	CHF *n* (%)	Controls *n* (%)	HWE *p* ^a^	Dominant Model *p* ^a^	Recessive Model *p* ^a^	Additive Model *p* ^a^	Allele Contrast *p* ^a^
rs762539							
TT (W)	140 (46.82)	198 (49.75)	0.46	0.44	0.73	0.62	0.67
CT	132 (44.15)	161 (40.45)					
CC	27 (9.03)	39 (9.8)					
rs2007720							
AA (W)	175 (58.92)	243 (61.52)	0.88	0.49	0.88	0.79	0.53
AG	107 (36.03)	133 (33.67)					
GG	15 (5.05)	19 (4.81)					
rs6031678							
CC	234 (78.26)	319 (79.95)	0.82	0.59	0.43 ^b^	0.52	0.43
CT	59 (19.73)	76 (19.05)					
TT	6 (2.0)	4 (1)					
rs8119756							
AA	223 (74.83)	270 (68.01)	0.82	0.04996	0.47	0.143	0.051
AG	68 (22.82)	114 (28.72)					
GG	7 (2.35)	13 (3.27)					
rs244072							
TT	205 (68.56)	272 (68.34)	0.39	0.95	0.59	0.86	0.81
TC	85 (28.43)	111 (27.89)					
CC	9 (3.01)	15 (3.77)					
rs6031689							
TT	228 (76.25)	320 (80.4)	0.08	0.19	0.74	0.33	0.27
GT	66 (22.07)	70 (17.59)					
GG	5 (1.67)	8 (2.01)					
rs2299694							
TT	134 (44.97)	189 (47.61)	0.44	0.49	0.71	0.78	0.48
TC	129 (43.29)	165 (41.56)					
CC	35 (11.74)	43 (10.83)					
rs452159							
CC	93 (31.31)	162 (40.7)	0.14	0.011	0.48	0.039	0.032
CA	151 (50.84)	173 (43.47)					
AA	53 (17.85)	63 (15.83)					
rs73598374							
GG	259 (87.21)	361 (90.48)	0.046	0.17	0.83^b^	0.23	0.26
AG	37 (12.46)	35 (8.77)					
AA	1 (0.34)	3 (0.75)					

Abbreviations: CHF, chronic heart failure; HWE, Hardy–Weinberg equilibrium; ^a^ *p* values was calculated from two-sided χ^2^-test.

**Table 3:** Logistic regression analysis for rs452159 and rs8119756 under different contrast models adjusted for age, sex, and cardiovascular risk factors.

SNPs	Dominant Model	Recessive Model	Additive Model	Allele Contrast
	Odds Ratio (95% CI) *p*	Odds Ratio (95% CI) *p*	Odds Ratio (95% CI) *p*	Odds Ratio (95% CI) *p*
rs452159	1.537 (1.10–2.16) 0.013	1.005 (0.65–1.55) 0.981	1.224 (0.97–1.54) 0.085	1.229 (0.98–1.55) 0.081
rs8119756	0.775 (0.54–1.10) 0.164	0.912 (0.34–2.47) 0.856	0.816(0.60–1.12) 0.204	0.814 (0.59–1.12) 0.201

### 2.3. Association between Genotype Distribution and Allele Frequency and Clinicopathological Parameters in Patients with CHF

The genotype distribution and allele frequency of the rs452159 did not differ significantly with functional New York Heart Association class (NYHA). However, the prevalence of the rs452159 AA genotype and A allele were significantly higher in CHF dyslipidemia (Table 4).

**Table 4:** Rs452159 polymorphisms genotype distribution and allele frequency according to NYHA and subsets of CHF patients.

Genotype	NYHA			Hypertension *n* = 172	Dyslipidemia *n* = 84	Diabetes *n* = 104	Smoking Habit *n* = 91
	II	III	IV				
rs452159							
CC	28 (30.1%)	33 (28.45%)	32 (40.51%)	57 (33.52%)	18 (21.43%)	32 (30.77%)	25 (28.41%)
CA	46 (49.46%)	59 (50.86%)	46 (58.23%)	77 (45.29%)	44 (52.38%)	50 (48.08%)	46 (52.27%)
AA	19 (20.43%)	24 (20.69%)	10 (12.66%)	36 (21.18%)	22 (26.19%)	22 (21.15%)	17 (19.32%)
Allele frequency (*p*-value)	0.18 ^a^			0.751 ^b^	0.0049 ^b^	0.487 ^b^	0.485 ^b^
Genotype distribution (*p*-value)	0.395 ^a^			0.064 ^b^	0.016 ^b^	0.539 ^b^	0.764 ^b^

^a^ χ^2^-test: allele frequency and genotype distribution among the three groups; ^b^ χ^2^-test: allele frequency and genotype distribution *vs.* controls.

The role of the rs452159 was further analyzed relative to parameters related to the severity of CHF, such as left ventricular ejection fraction (LVEF), left-ventricle end-diastolic diameter (LVEDD), and left-ventricle end-systolic diameter (LVESD). No association was found between any of these cardiovascular parameters and genotype for the rs452159 variant (Table 5).

**Table 5:** Parameters related to the severity of CHF in different genotype groups.

Genotype	LVEF Mean Value	LVEDD Mean Value	LVESD Mean Value
rs452159			
CC	42.14 ± 14.08	64.34 ± 11.71	51.39 ± 13.56
CA	39.39 ± 12	66.22 ± 10.75	53.70 ± 12.43
AA	42.42 ± 13.33	63.77 ± 10.91	51.02 ± 12.14
*p* ^a^	0.165	0.260	0.254

Abbreviations: LVEF, left ventricular ejection fraction; LVEDD, left-ventricle end-diastolic diameter; LVESD, left-ventricle end-systolic diameter. multiple comparisons among pairs of means were also tested using Dunnett’s test and no significance was found; ^a^ *p* values was calculated from two-sided Analysis of Variance.

## 3. Discussion

This is the first study to evaluate the effects of ADA gene SNPs as potential predisposing factors to CHF. The results of this study provide evidence for the role of *ADA* SNP in predisposing the patient to CHF independent of cardiovascular risk factors, but not in modulating the severity of the disease.

We analyzed the association under four common contrast models. After logistic regression, none of the nine SNPs was associated with CHF risk under the recessive model which assumed a recessive effect of the minor allele of each SNP; under the additive model, assuming that there is a linear gradient in risk among the homozygous, heterozygous, and wild type genotypes, no association was found for any SNP; under the allele contrast model (variant allele *vs.* wild allele), similar results were found; under the dominant model, assuming a dominant effect of the minor allele of each SNP, rs452159 was found to be associated with CHF risk, while, other SNPs were not. In other words, comparing with other genotypes, rs452159 CC genotype might be a protective factor for CHF.

The SNP found to be associated with CHF in this study was located in intron areas of the ADA gene, and therefore did not, in theory, cause a change in the encoding protein. However, Choi *et al.* indicated that it is possible for an intronic SNP to alter the splicing of primary transcripts or gene expression. Those authors demonstrated that rs2280964, which is an intronic SNP, affected the alternative splicing of the chemokine receptor CXCR3 [15]. Many other studies have also revealed that specific subsets of exonic splicing silencers exerted distinct effects on a multifunctional intron retention reporter, and that one of these subsets is likely preferred for regulation of endogenous intron retention events. Intron retention could therefore affect alternative splicing of pre-mRNA by influencing the exonic splicing silencers. Through alternative splicing, a gene could produce different mature mRNAs and then translated proteins with different functions, and in extreme cases with distinct functions and sometimes opposing functions [16,17,18]. Therefore, in the present study we selected the SNP associated with CHF from the statistics perspective; this SNP may also have an impact on protein function. Further studies are thus needed to ascertain the biological function of the proteins encoded by this SNP.

The most widely studied SNP within the ADA gene is 22G>A (rs73598374), which is a G-to-A transition at nucleotide 22 in exon 1 that results in an Asp-to-Asn alteration [19]. The role of this SNP has been widely researched. Napolioni *et al.* found that 22G>A was significantly associated with human life expectancy in males [20], and Dutra *et al.* found a significant decrease in the frequency of the G/A genotype in schizophrenic patients [21]. As to its impact on sleeping, it has been reported that in healthy adults this SNP distinctly affects non-rapid-eye-movement sleep intensity, electroencephalogram θ/α frequencies in sleep and wakefulness, attention, subjective sleepiness, and salivary α-amylase activity [22]. A similar conclusion was drawn by Mazzotti *et al.*, who suggested that the *ADA* G22A polymorphism is an important source of variation in sleep homeostasis in humans [23]. Regarding its effect on the heart, Safranow *et al.* concluded that the A allele may decrease genetic susceptibility to coronary artery disease [24]. In contrast, from *in vivo* study, Riksen *et al.* concluded that although heterozygosity for the 22G>A variant of ADA reduces catalytic activity, it does not enhance forearm reactive hyperemia; in other words, the 22G>A variant is unlikely to contribute to any variability in the protective cardiovascular effects of Ado [25]. These contradictory results show that further research is needed to elucidate the role of this SNP on the heart. In the present study, we were unable to locate this SNP in the Chinese Han population through the HapMap database, indicating that the minor allele frequency of this SNP in this population was very low. Since the A-allele frequency was particularly low among our 300 CHF patients and 400 controls, we decided not to study it further.

The rs452159 variant was found to be a risk factor for CHF in the present study. Pangilinan *et al.*, who investigated the association between 82 candidate genes and neural-tube defects, found that this SNP was significantly and strongly associated with maternal risk in a dominant model of logistic regression [26]. Gass *et al.* also demonstrated that this SNP was associated with depression and fatigue in men [27].

The present study is the first to demonstrate that the rs452159 is associated with susceptibility to CHF. The present findings suggest the importance of genotype testing, since it can be used to identify people who are carrying the risk genotypes that would render them more susceptible to CHF. If identified, such people will be able to take the necessary protective measures, which are particularly important for those with traditional cardiovascular risk factors. In future studies, we plan to verify the function of the SNP from the biological perspective to clarify the mechanism underlying the effects of this SNP on CHF.

The metabolism of Ado in patients with CHF remains to be fully elucidated, although the authors of one study suggested that the destruction of the Ado-related signal transduction system could be one of the reasons for heart failure; cyclic Ado levels are increased while Ado receptors are down-regulated in patients with heart failure [28]. It has also been found that this increase in Ado levels was related to the up-regulation of 5'-nucleotidase and the down-regulation of ADA [28,29]. However, Del *et al.* studied the expression of ADA in normal and failing minipig hearts, and found a trend toward a higher expression of mRNA Ado deaminase in the myocardium of pigs with heart failure [5]. The expression of ADA in heart failure has not yet been determined, although it is certain that ADA activity is one of the important factors affecting Ado levels and that it participates in the process of heart failure. The association between the SNP selected in the present study with CHF verifies that point.

We were unable to demonstrate the influence of *ADA* locus on the severity of the disease, since the allele frequency and genotype distribution did not differ with NYHA class, EF, LVEDD, or LVESD. These data possibly strengthen the observation that the *ADA* locus could be involved in the cardioprotective activity of Ado in CHF, but not in the disease progression.

In this study, we also found that rs452159 was associated with CHF dyslipidemia, which suggested that this SNP might exert certain influence on dyslipidemia. There was excellent evidence that adenosine could regulate several aspects of adipose tissue function including lipolysis [30,31]. *ADA*, as the most important enzyme for adenosine metabolism, was also related to lipid metabolism [32,33,34]. The study of Lin *et al.* found that the activity of ADA in high fat diet group was higher than in the normal diet group [33]. Bottini *et al.* also found that low ACP1 activity/high ADA activity joint was positively associated with dyslipidemia [34]. These findings, consistent with ours, showed the role of *ADA* on the lipid metabolism.

The limitations of this meta-analysis should be considered when interpreting its findings. First, Ado is produced or degraded by several enzymes, including 5'-nucleotidase, ADA, and Ado kinase [11]. One limitation of the present study is the lack of a more comprehensive genetic analysis of the polymorphisms potentially associated with Ado levels. Second, we were unable to establish the mechanisms underlying the effects of the two selected SNPs on CHF.

## 4. Experimental Section

### 4.1. Subjects

Chinese patients aged >18 years with CHF at the First Affiliated Hospital, College of Medicine, Xi’an Jiaotong University (Xi’an, China) in 2013 and 2014 were enrolled. The main inclusion criterion was diagnosis of heart failure according to the Guidelines for Diagnosis and Treatment of Heart Failure in China (2013). The cause of heart failure was determined in each patient by clinical assessment and echocardiography. The exclusion criteria were age <18 years and the presence of severe hepatic or renal insufficiency, tumors or malignant disease, acute attack of CHF or severe acute infection. Patients who suffered acute myocardial infarction but who were not revascularized within 2 weeks were also excluded. All sample selections were completed according to strict inclusion criteria by two visiting staff members and were reviewed by the Chief Physician of Cardiology at our institution. Ultimately, 300 patients were eligible for inclusion in this study.

The control group comprised 400 sex- and ethnicity-matched healthy volunteers (age > 18 years old) at the Medical Examination Center of the same hospital who had no known personal and/or family history of cardiovascular disease. Each of the participants submitted to a detailed interview in which the details of their personal and familial history were obtained in the framework of a physical examination by expert physicians, in order to identify symptom-free subjects and to exclude those who were suspected of having any form of vascular disease.

Demographic and clinical characteristics (mainly including gender, age, and traditional cardiovascular risk factors) were obtained via interviewer-administered health-risk questionnaires. The subjects were considered to have hypertension according to Guidelines for Prevention and Treatment of Hypertension in China (2013), or if they were taking antihypertensive drugs. Dyslipidemia was defined according to the Guidelines for Prevention and Treatment of Blood Lipid Abnormality in Chinese Adults (2007), and diabetes was defined according to the criteria of Guidelines for Prevention and Treatment of diabetes in China (2013). Information about smoking status, were self-reported. Individuals were designated “smokers’’ if they have smoked ≥5 cigarettes a day for ≥12 months [35].

The study protocol was drawn up in compliance with the principles of the Helsinki Accord, and was reviewed and approved by the medical ethics committee of the First Affiliated Hospital of Medical College, Xi’an Jiaotong University (No. 2014042; 1 March 2014). Statement of informed consent was obtained from all participants after a full explanation of the procedure.

### 4.2. Genotyping Assay

The blood samples were collected into tubes containing Ethylene Diamine Tetraacetic Acid. After centrifugation, the samples were stored at −80 °C until analysis. Standard phenol–chloroform extraction method was used to extract genomic DNA from whole blood. DNA concentration was measured by spectrometry (DU530 UV/VIS spectrophotometer, Beckman Instruments, Fullerton, CA, USA). Eight tag SNPs (*i.e.*, rs762539, rs2007720, rs6031678, rs8119756, rs244072, rs6031689, rs2299694 and rs452159) that captured most of the known common *ADA* variations according to Chinese Han population data from HapMap [36], and one SNP (rs73598374) yielded by a literature search were selected for the present study. Sequenom MassARRAY Assay Design 3.0 software was used to design a Multiplexed SNP MassEXTEND assay [37,38,39]. SNP genotyping was performed using the Sequenom MassARRAY RS1000 system (Sequenom Inc., San Diego, CA, USA) according to the standard protocol recommended by the manufacturer [39]. The primers used for each SNP in the present study are listed in Table 6. Sequenom Typer 4.0 software (Sequenom Inc., San Diego, CA, USA) was used for data management and analyses [37,38,39,40].

**Table 6:** Primers used for this study.

SNP_ID	Allele	1st-PCRP (5'–3')	2nd-PCRP (5'–3')	UEP_SEQ (5'–3')
rs762539	T/C	ACGTTGGATGACTTTT ATCTCACGGAATC	ACGTTGGATGTCCACT CCCTAAGCTTCAGT	GCCATGCCTTGT CAAGTC
rs2007720	A/G	ACGTTGGATGTGCAG GGACCCAAAATCTTG	ACGTTGGATGTACCAG GTGCCTTCAGTGAC	GGCCTTCAGTGA CTTTTTCTT
rs6031678	C/T	ACGTTGGATGGGGTTT GGGAGTATGGTATC	ACGTTGGATGTTGTCT TGGACTGTTGAGGC	CTCCAAAGATTC CAGGCCC
rs8119756	A/G	ACGTTGGATGCTTCCT CTTCTTACCTCCAC	ACGTTGGATGTAGAGG AACCGTTCTAGAGG	AACGGAGTGAG GGTAGAATCC
rs244072	T/C	ACGTTGGATGTTGGAT GCTTGGACCTCCTG	ACGTTGGATGAATCCT CCACAAAGTAGAAC	AGATGATGAAAAA TAGGAGTAAAC
rs6031689	T/G	ACGTTGGATGGTGAG AAGGGATGAGTGCTA	ACGTTGGATGTAGGGG TTCAGGGAGCTGG	GGGAAGAGCCA GCTGCCCACCA
rs2299694	T/C	ACGTTGGATGCCGGGT TAAGTTATTGAAGC	ACGTTGGATGTTACTC CACCTACCACGGCT	GAACGGCAATAG AGTTCCT
rs452159	C/T	ACGTTGGATGGTGTGT TGGGAAAAGATCAC	ACGTTGGATGACACTA AGCACACGCAGCTC	GATCATTCTTTCT TCTCCCTGG
rs73598374	G/A	ACGTTGGATGCCCCGC GCGCGCTCACTTT	ACGTTGGATGACGAG GGCACCATGGCCCAG	TCCCAGACGCCC GCCTTC

### 4.3. Statistical Analysis

Statistical analysis was performed using SPSS 18.0 for Windows (PASW Statistics, SPSS Inc., Chicago, IL, USA). The χ^2^-test was used to test for deviation of the genotype distribution from Hardy–Weinberg equilibrium (HWE). Differences in genotype and allele frequencies between study groups were also estimated by χ^2^-test. In addition, the association between *ADA* SNPs and CHF was assessed using logistic regression analysis under dominant, recessive, and additive genetic models.

Differences among the different genotype groups with respect to parameters related to the severity of CHF, such as ejection fraction (EF), left-ventricle end-diastolic diameter (LVEDD), and left-ventricle end-systolic diameter (LVESD), were estimated by analysis of variance, and the significant differences among pairs of means were tested using Dunnett’s test. Differences in the genotype distribution and allele frequency for the significant SNP according to functional New York Heart Association (NYHA) class were also analyzed using the χ^2^-test. The cutoff for statistical significance was set at *p* < 0.05 (two-sided).

## 5. Conclusions

In summary, to the best of our knowledge, this is the first report investigating an association between ADA gene polymorphism and the risk and progress of CHF in Chinese patients. Evidences have been found from this preliminary study that rs452159 in *ADA* is a risk factor for CHF. This conclusion may provide a novel insight into the contribution of Ado to heart function.
